# Contributions to the knowledge of Albanian Chrysomelidae (Coleoptera), with the redescription of *Luperus flaviceps* Apfelbeck, 1912

**DOI:** 10.3897/zookeys.1285.189385

**Published:** 2026-07-21

**Authors:** Giulia Magoga, Alevtina Andreeva, Paola Costagliola, Marilù Cardinale, Matteo Brunetti, Matteo Montagna

**Affiliations:** 1 Department of Agricultural Sciences, University of Naples Federico II, Piazza Carlo di Borbone 1, 80055 Portici, Italy Department of Biosciences, University of Milan Milan Italy; 2 Department of Insect Symbiosis, Max Planck Institute for Chemical Ecology, 07745 Jena, Germany Department of Insect Symbiosis, Max Planck Institute for Chemical Ecology Jena Germany; 3 Via Francesco De Pinedo 7, 80144 Naples, Italy Department of Agricultural Sciences, University of Naples Federico II Portici Italy; 4 Department of Biosciences, University of Milan, Via Celoria 26, 20133 Milan, Italy Unaffiliated Naples Italy

**Keywords:** Balkan Peninsula, DNA barcoding, fauna of Albania, leaf beetles, molecular identification, new species

## Abstract

The Balkan region is among the least-studied areas in Europe with respect to Chrysomelidae, particularly over the last decades. This study aims to contribute new data to the knowledge of the Albanian chrysomelid fauna. Specifically, it aims to: (i) provide new information on the distribution of chrysomelid species within the country; (ii) develop a reference library of DNA barcode sequences for the molecular identification of Albanian Chrysomelidae; and (iii) redescribe *Luperus flaviceps* Apfelbeck, 1912, a rare chrysomelid found in Albania whose original description is brief and lacks some key diagnostic characters. Chrysomelidae were collected from multiple localities in central and southern Albania. As a result, 12 species are reported from Albania for the first time, together with three additional taxa that likely represent species new to science. Furthermore, updated distributional data for 81 species within the country are provided. A total of 71 DNA barcode sequences representing 57 species were generated; they constitute the only publicly available barcode sequences for Albanian Chrysomelidae, and eight of them represent new taxon records for public reference databases. The redescription of *L. flaviceps* is provided, including high-quality photographs of both males and females, as well as a detailed description and illustration of the aedeagus morphology, which was previously unknown. The results presented here emphasize the need for further investigations on Albanian Chrysomelidae to improve our understanding of their diversity within the country.

## Introduction

Chrysomelidae (Coleoptera) is one of the most species-rich families of phytophagous insects (Slipinski et al. 2011) and have a particularly high species diversity documented in the Palaearctic region (Bezděk and Sekerka 2024). In Europe, approximately 1,800 species are present (Audisio et al. 2015). Owing to their remarkable taxonomic diversity, ecological significance, and, for some species, economic importance, Chrysomelidae have been the subject of numerous taxonomic and faunistic studies. Despite this long tradition of study, the level of faunistic knowledge remains uneven across Europe. In particular, some areas of the Balkan Peninsula are still poorly investigated (Gruev 1992; Vig 2002; Gavrilović and Ćurčić 2011; Gavrilović et al. 2014). While neighbouring countries such Bulgaria (Warchałowski 1974, 1976; Borowiec 1979; Gruev and Tomov 1984, 1986, 1998, 2007; Vig 1992; Gruev 2004, 2006; Bechev and Pavlova 2016), Greece (Doguet 1988; Gruev 1990, 1992; Vig 2003; Rozner and Rozner 2014), and North Macedonia (Gruev 1998; Rozner and Rozner 2008) have been relatively well studied, Albania remains one of the least investigated territories in the Balkans. The first comprehensive studies of the Albanian leaf beetle fauna, conducted as part of research on the entire Balkan Peninsula, were initiated at the end of the 19^th^ century by Viktor Apfelbeck (1912, 1914, 1916) and were later supplemented by the faunistic records of Jan Roubal (Roubal 1931–1934). Apfelbeck summarized the existing literature and provided a significant contribution to the knowledge of Chrysomelidae. Following these early contributions, historical national surveys remained scarce, primarily consisting of Csiki’s expedition (Csiki 1940) and the Albanian Expedition of the German Entomological Institute in 1961 (Mohr 1965, 1966), which together provided the fundamental baseline for the knowledge of Albanian Chrysomelidae. These foundational data were subsequently incorporated into broader regional syntheses and catalogues, including the Palaearctic treatments of Alticini (Gruev and Döberl 1997, 2005) and the monograph on European and Mediterranean Chrysomelidae (Warchałowski 2003), as well as broader historical compilations for the Balkan Peninsula, which include a faunistic list for Albania (Gruev 2005). The latest edition of the Catalogue of Palaearctic Coleoptera (Bezděk and Sekerka 2024), currently the most comprehensive and up-to-date reference for the Albanian Chrysomelidae, lists 358 species distributed across the following subfamilies: Donaciinae (8 spp.), Criocerinae (10 spp.), Chrysomelinae (50 spp.), Galerucinae (152 spp.), Cryptocephalinae (60 spp.), Cassidinae (22 spp.), Eumolpinae (8 spp.), Bruchinae (48 spp.). Nevertheless, considering that Albania is a Mediterranean country characterized by pronounced environmental heterogeneity, including strong climatic contrasts between coastal and inland areas, marked altitudinal gradients, and a wide diversity of habitat types, the currently known species richness appears low, particularly when compared with neighbouring countries within the same biogeographical region (as reflected by species occurrences per country reported by Bezděk and Sekerka (2024)).

In recent years, DNA barcoding, using a fragment of the mitochondrial cytochrome c oxidase subunit I (COI) gene as a marker, has become a crucial tool for species level identification of metazoans (Hebert et al. 2003, 2004). It significantly accelerates identification procedures, particularly for taxa in which morphological identification is difficult and requires specialized taxonomic expertise, such as insects (Magoga et al. 2022). However, accurate species-level identification using DNA barcoding relies on the availability of comprehensive and reliable reference sequence data. At present, the representation of Balkan leaf beetle barcode sequences in major public databases used as reference for the molecular identification, i.e. Barcode of Life Data System (BOLD, https://boldsystems.org/) (Ratnasingham and Hebert 2007) and GenBank (Clark et al. 2016), remain very limited. Notably, no chrysomelid records from Albania are currently available in them (data updated to January 2026), highlighting a substantial gap in reference barcodes for this country.

This study aims to contribute with new data to the knowledge of the Albanian chrysomelid fauna. In particular, it aims to: (i) provide new information on the distribution of chrysomelid species within the country (excluding Bruchinae); (ii) develop a reference library of DNA barcode sequences for the molecular identification of Albanian Chrysomelidae (excluding Bruchinae); and (iii) provide the redescription of *Luperus flaviceps* Apfelbeck, 1912, a rare species occurring in Albania.

## Methods

### Specimen collection and morphological identification

While recent phylogenetic and taxonomic studies have refined the concept of Chrysomelidae to include Bruchinae, and historically this group has been treated as a separate family (Bruchidae) due to their seed-feeding biology and distinctive morphology, this subfamily was excluded from the sampling in the present study. This decision was based on the authors’ limited experience in reliably identifying members of this group to species level, and on the intention to focus on taxa that can be identified morphologically with sufficient confidence and accuracy. The specimens included in this study were collected using sweep nets during a field campaign conducted during summer 2025 (24 June–1 July) in the Albanian counties of Elbasan, Korçë, Berat, Gjirokastër and Vlorë. Collected specimens were preserved in 98% ethanol and stored at 4 °C until morphological examination. Morphological identification was carried out using a ZEISS Stemi 305 stereomicroscope (ZEISS, Germany), based on the examination of both external characters and genitalia. Species-level identifications were achieved using dichotomous keys (Müller 1953; Burlini 1955; Mohr 1981; Doguet 1994; Konstantinov 1998; Warchałowski 2010). When necessary, additional taxonomic literature specific to particular species groups was consulted. The nomenclature and taxonomic circumscription of Chrysomelidae followed in this study are those of Bezděk and Sekerka (2024), thus accordingly, Megalopodidae and Orsodacnidae are considered as separate families.

Comparative specimens from specialist dry collections of Chrysomelidae were examined to support and confirm identifications. These reference collections included those of Matteo Montagna, Davide Sassi, and Andrzej Bieńkowski. Occurrence records for the detected taxa were registered in the Global Biodiversity Information Facility (GBIF; https://doi.org/10.15468/h2pfar).

*Luperus flaviceps* was identified following the dichotomous key of Warchałowski (2010) and through direct comparison of the collected specimens with conspecific material from Albania housed in the Jan Bezděk collection. In addition, high-quality photographs of the type specimens provided by the National Museum of Bosnia and Herzegovina were examined by the authors.

### Generation of reference DNA barcodes

Reference DNA barcode sequences were generated for all recorded species, except those that are very common in Europe, very easy to be morphologically identified (following the criteria of Magoga et al. 2021), and already well represented in reference databases. However, all species representing new records for Albania were barcoded, including those that are otherwise common and already well represented in reference databases. Following morphological identification, specimens selected for barcoding were processed according to their size. Specimens smaller than 2 mm were dissected to separate the head-thorax from the abdomen, whereas in specimens larger than 2 mm, a single leg was removed. All specimens were air-dried under sterile airflow until complete ethanol evaporation prior to DNA extraction. DNA extraction was carried out using the Wizard® Genomic DNA Purification Kit (Promega, USA). For specimens from which the head-thorax and abdomen were separated, DNA was extracted in a non-destructive manner by immersing the body parts in 600 µL of Nuclei Lysis Solution supplemented with proteinase K (200 ng/mL; Sigma-Aldrich, USA). In contrast, removed legs were mechanically crushed using sterile pestles in the same solution. Samples were incubated at 56 °C for 3 h, and the remaining steps of the extraction protocol were carried out according to the manufacturer’s instructions of Wizard® Genomic DNA Purification Kit. Extracted DNA was used as template for the amplification of the barcode region (a fragment of the mitochondrial COI gene) by PCR, using LCO1490–HCO2198 primer pair (Folmer et al. 1994). When this pair of primers resulted in unsuccessful amplification of the target region, other primers amplifying the same gene region were used, i.e. LepF1 5' - ATT CAA CCA ATC ATA AAG ATA TTG G / LepR1 5' - TAA ACT TCT GGA TGT CCA AAA AAT CA (Hebert et al. 2004). PCRs conditions used follow Andreeva et al. (2025). PCR products were visualized by 1.5% agarose gel electrophoresis, and positive amplicons were directly sequenced on forward strands using Sanger sequencing (Microsynth, Switzerland). Electropherograms were inspected using Geneious Prime v. 2025.0.3 (MM license), and the open reading frame in the obtained sequences was verified using the online tool EMBOSS Transeq (http://www.ebi.ac.uk/Tools/st/emboss_transeq/). To validate the generated barcode sequences, they were compared with reference sequences available in the two major databases used for insect molecular identification: the Barcode of Life Data System (BOLD; Ratnasingham and Hebert 2007) and GenBank (Clark et al. 2016) (updated to January 2026). Comparisons in BOLD were performed using the BOLD identification engine on animal public library with the exhaustive search as operating mode. Morphological identification was considered congruent with molecular identification when the best match of the query sequence was a reference sequence labelled with the same species name and showed a sequence similarity ≥ 96%. For GenBank comparisons, the Basic Local Alignment Search Tool (BLAST; http://www.ncbi.nlm.nih.gov/BLAST; Altschul et al. 1990) search was performed using default parameters. Congruence between morphological and molecular identification was accepted only when the query and reference sequences had a sequence identity ≥ 96% and an E-value < 1 × 10^−20^. In cases where morphological and molecular identifications were incongruent, specimen morphology was re-examined. If the discrepancy could not be attributed to an error in morphological identification, the morphological identification was retained as the valid identification, and the corresponding barcode was labelled accordingly. This approach was adopted because reference databases contain chrysomelid sequences derived from misidentified specimens, which can bias molecular identification results (Salvi et al. 2020). Additionally, in some cases, DNA barcoding fails as a tool for chrysomelid species identification (Magoga et al. 2016, 2018). Finally, processed specimens were mounted on pins together with their genitalia and dry preserved. Generated barcode sequences were deposited in BOLD (IDs: MEDLB970-26–MEDLB1040-26), together with the voucher specimen images.

## Results

### Morphological identification

**Figure 1.**
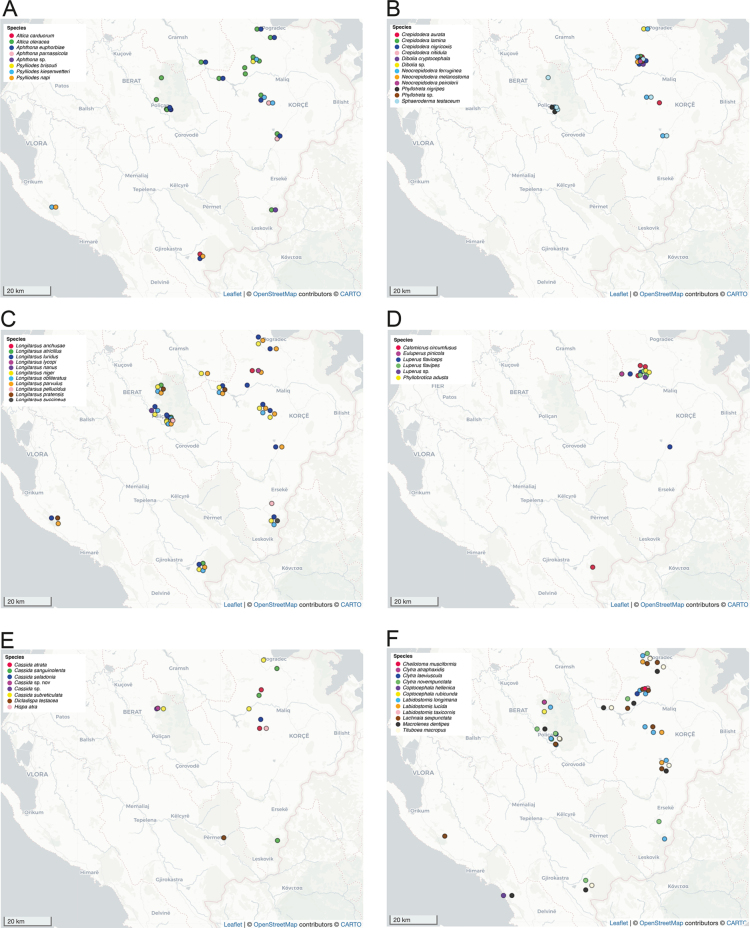
Albanian localities where species of Chrysomelidae were recorded. **A**. Galerucinae: Alticitae, genera *Altica*, *Aphthona*, and *Psylliodes*; **B**. Galerucinae: Alticitae, genera *Crepidodera*, *Dibolia*, *Neocrepidodera*, *Phyllotreta*, and *Sphaeroderma*; **C**. Galerucinae: Alticitae, genus *Longitarsus*; **D**. Galerucinae: Galerucinae; **E**. Cassidinae; **F**. Cryptocephalinae: Clytrini, genera *Cheilotoma*, *Clytra*, *Coptocephala*, *Labidostomis*, *Lachnaia*, *Macrolenes*, and *Tituboea*.

**Figure 2.**
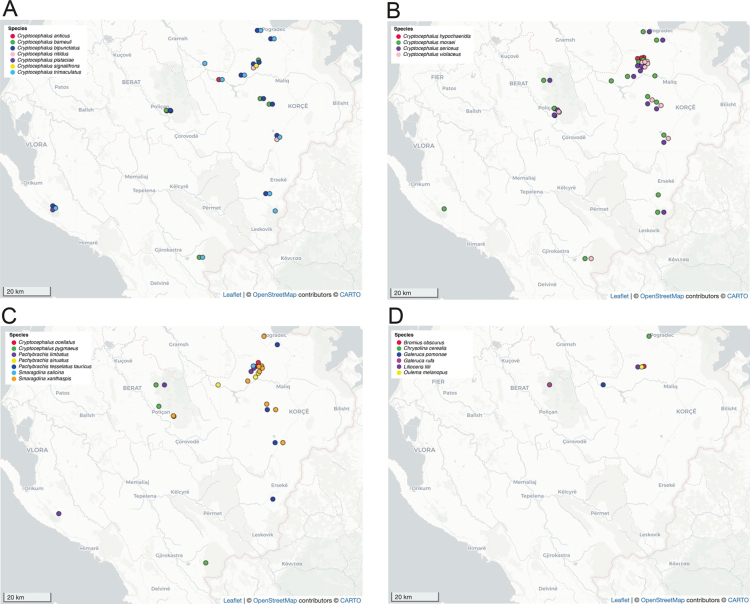
Albanian localities where species of Chrysomelidae were recorded. **A**. Cryptocephalinae: Cryptocephalini, genus *Cryptocephalus* (part 1); **B**. Cryptocephalinae: Cryptocephalini, genus *Cryptocephalus* (part 2); **C**. Cryptocephalinae: Cryptocephalini, genus *Cryptocephalus* (part 3), Cryptocephalinae: Pachybrachini, genus *Pachybrachis*; Cryptocephalinae: Clytrini, genus *Smaragdina*; **D**. Other Chrysomelidae: Eumolpinae: Adoxini; Chrysomelidae: Chrysomelini; Galerucinae: Galerucinae; Criocerinae.

During the collection campaign conducted in central and southern Albania, a total of 2006 chrysomelid specimens were recorded from 29 collection points (Table 1, Suppl. material 1, Figs 1, 2). Based on morphological examination, these specimens were assigned to 81 species (Table 1), mainly belonging to the subfamilies Cryptocephalinae and Galerucinae, as expected, since members of these subfamilies are typically more abundant than others during the period in which the sampling campaign was conducted. For six of them, identification could be achieved only to the genus level. Although they were clearly distinguishable as distinct morphotypes from the other collected species, species-level identification was not possible for one of the following reasons: specimens were immature and their genitalia could not be examined (*Cassida* sp.); specimens belonged to a sex for which external and genital morphology is not diagnostic for species identification (*Luperus* sp., *Phyllotreta* sp.); and specimens exhibited morphological characters that differ from all currently described species within their respective genera, suggesting that they may represent species new to science (*Cassida* sp. nov., *Dibolia* sp., *Aphthona* sp.). All these taxa were represented by a single specimen, except *Luperus* sp., for which two specimens were collected (Table 1, Suppl. material 1).

**Table 1.** Chrysomelidae recorded in Albania. For each species, the number of individuals recorded, the BOLD ID of the generated barcode sequences (when available), and whether the species represents a new record for the Albanian fauna or for public DNA barcode reference databases are reported. The subfamily to which each species belongs is also provided as a note.

**Species**	**N. ind.^a^**	**Barcode IDs**	**New Albania^b^**	**New datab.^c^**
*Altica carduorum* Guérin-Méneville, 1858^ga^	16	MEDLB1013-26		
*Altica oleracea* (Linnaeus, 1758)^ga^	257	MEDLB970-26		
*Aphthona euphorbiae* (Schrank, 1781)^ga^	74			
*Aphthona parnassicola* Heikertinger, 1944^ga^	2	MEDLB1001-26	X	X
		MEDLB1012-26		
*Aphthona* sp.^ga^	1			
*Calomicrus circumfusus* (Marsham, 1802)^ga^	13	MEDLB971-26	X	
*Crepidodera aurata* (Marsham, 1802)^ga^	7			
*Crepidodera lamina* (Bedel, 1901)^ga^	4	MEDLB1017-26	X	
*Crepidodera nigricoxis* Heikertinger, 1912^ga^	17	MEDLB1000-26		
*Crepidodera nitidula* (Linnaeus, 1758)^ga^	2	MEDLB976-26	X	
*Dibolia cryptocephala* (Koch, 1803)^ga^	1			
*Dibolia* sp.^ga^	1	MEDLB1005-26		X
*Euluperus pinicola* (Duftschmid, 1825)^ga^	1			
*Galeruca pomonae* (Scopoli, 1763)^ga^	1			
*Galeruca rufa* Germar, 1824^ga^	1	MEDLB988-26		
*Longitarsus anchusae* (Paykull, 1799)^ga^	5			
*Longitarsus atricillus* (Linnaeus, 1761)^ga^	27	MEDLB990-26		
		MEDLB1016-26		
*Longitarsus luridus* (Scopoli, 1763)^ga^	129	MEDLB1014-26		
*Longitarsus lycopi* (Foudras, 1860)^ga^	5	MEDLB991-26		
*Longitarsus nanus* (Foudras, 1860)^ga^	17	MEDLB1020-26	X	
*Longitarsus niger* (Koch, 1803)^ga^	14	MEDLB992-26		
*Longitarsus obliteratus* (Rosenhauer, 1847)^ga^	60			
*Longitarsus parvulus* (Paykull, 1799)^ga^	198	MEDLB1002-26		
		MEDLB993-26		
		MEDLB1022-26		
*Longitarsus pellucidus* (Foudras, 1860)^ga^	5	MEDLB1034-26		
*Longitarsus pratensis* (Panzer, 1794)^ga^	20	MEDLB1028-26		
		MEDLB1037-26		
		MEDLB1036-26		
		MEDLB1023-26		
*Longitarsus succineus* (Foudras, 1860)^ga^	2	MEDLB1021-26		
*Luperus flaviceps* Apfelbeck, 1912^ga^	7	MEDLB1010-26		X
		MEDLB1011-26		
*Luperus flavipes* (Linnaeus, 1767)^ga^	4			
*Luperus* sp.^ga^	2			
*Neocrepidodera ferruginea* (Scopoli, 1763)^ga^	31			
*Neocrepidodera melanostoma* (Redtenbacher, 1849)^ga^	25			
*Neocrepidodera peirolerii* (Kutschera, 1860)^ga^	1	MEDLB995-26	X	
*Phyllobrotica adusta* (Creutzer, 1799)^ga^	2	MEDLB1025-26		X
*Phyllotreta nigripes* (Fabricius, 1775)^ga^	13			
*Phyllotreta* sp.^ga^	1			
*Psylliodes brisouti* Bedel, 1898^ga^	2	MEDLB1018-26	X	
		MEDLB1035-26		
*Psylliodes kiesenwetteri* Kutschera, 1864^ga^	15			
*Psylliodes napi* (Fabricius, 1792)^ga^	2			
*Sphaeroderma testaceum* (Fabricius, 1775)^ga^	10			
*Cassida atrata* Fabricius, 1787^ca^	2	MEDLB972-26		X
*Cassida sanguinolenta* Müller, 1776^ca^	4	MEDLB996-26		
*Cassida seladonia* Gyllenhal, 1827^ca^	1	MEDLB1006-26		X
*Cassida* sp. nov.^ca^	1	MEDLB1038-26		X
*Cassida* sp.^ca^	1			
*Cassida subreticulata* Suffrian, 1844^ca^	26	MEDLB1007-26		
*Dicladispa testacea* (Linnaeus, 1767)^ca^	5			
*Hispa atra* Linnaeus, 1767^ca^	4			
*Cheilotoma musciformis* (Goeze, 1777)^cr^	2	MEDLB973-26	X	
*Clytra atraphaxidis* (Pallas, 1771)^cr^	2	MEDLB1008-26		
*Clytra laeviuscula* Ratzeburg, 1837^cr^	2			
*Clytra novempunctata* Olivier, 1808^cr^	14	MEDLB1003-26		
*Coptocephala hellenica* Desbrochers des L., 1870^cr^	9	MEDLB974-26		
*Coptocephala rubicunda* (Laicharting, 1781)^cr^	24	MEDLB975-26		
*Cryptocephalus anticus* Suffrian, 1848^cr^	1			
*Cryptocephalus bameuli* Duhaldeborde, 1999^cr^	16	MEDLB978-26		
		MEDLB979-26		
		MEDLB980-26		
		MEDLB981-26		
		MEDLB982-26		
		MEDLB1040-26		
*Cryptocephalus bipunctatus* (Linnaeus, 1758)^cr^	52	MEDLB983-26		
*Cryptocephalus hypochaeridis* (Linnaeus, 1758)^cr^	14			
*Cryptocephalus moraei* (Linnaeus, 1758)^cr^	109			
*Cryptocephalus nitidus* (Linnaeus, 1758)^cr^	2	MEDLB984-26	X	
*Cryptocephalus ocellatus* Drapiez, 1819^cr^	5	MEDLB999-26		
*Cryptocephalus pistaciae* Suffrian, 1854^cr^	1	MEDLB1039-26		
*Cryptocephalus pygmaeus* Fabricius, 1792^cr^	11	MEDLB1015-26		
*Cryptocephalus sericeus* (Linnaeus, 1758)^cr^	120	MEDLB977-26		
*Cryptocephalus signatifrons* Suffrian, 1847^cr^	1	MEDLB985-26		
*Cryptocephalus trimaculatus* Sharma, 1801^cr^	34	MEDLB986-26		
		MEDLB1009-26		
*Cryptocephalus violaceus* Laicharting, 1781^cr^	48	MEDLB987-26		
*Labidostomis longimana* (Linnaeus, 1760)^cr^	187	MEDLB1030-26		
*Labidostomis lucida* (Germar, 1824)^cr^	14	MEDLB1029-26	X	
		MEDLB1031-26		
*Labidostomis taxicornis* (Fabricius, 1792)^cr^	1	MEDLB1033-26	X	
*Lachnaia sexpunctata* (Scopoli, 1763)^cr^	36	MEDLB1032-26		
*Macrolenes dentipes* (Olivier, 1808)^cr^	60	MEDLB994-26		
*Pachybrachis limbatus* (Menetries, 1836)^cr^	24	MEDLB997-26		X
*Pachybrachis sinuatus* (Mulsant & Rey, 1859)^cr^	12	MEDLB998-26		
*Pachybrachis tesselatus tauricus* Suffrian, 1848^cr^	23	MEDLB1024-26		
*Smaragdina salicina* (Scopoli, 1763)^cr^	1			
*Smaragdina xanthaspis* (Germar, 1824)^cr^	117	MEDLB1026-26		
*Tituboea macropus* (Illiger, 1800)^cr^	14	MEDLB1027-26		
*Chrysolina cerealis* (Linnaeus, 1767)^ch^	1	MEDLB1004-26		
*Lilioceris lilii* (Scopoli, 1763)^ci^	1	MEDLB989-26		
*Oulema melanopus* (Linnaeus, 1758)^ci^	1			
*Bromius obscurus* (Linnaeus, 1758)^eu^	8	MEDLB1019-26	X	

Notes: ^a^ Number of individuals recorded; ^b^ New species records for Albania; ^c^ New species records for public reference databases of DNA barcode sequences. Subfamilies: ^ga^Galerucinae; ^ca^Cassidinae; ^cr^Cryptocephalinae; ^ch^Chrysomelinae; ^ci^Criocerinae; ^eu^Eumolpinae.

### Albanian Chrysomelidae DNA barcode library

A total of 78 collected specimens were processed for DNA barcode sequence generation. For seven specimens, PCR amplification of the target region was unsuccessful despite the use of both selected primer pairs (*Cryptocephalus anticus* Suffrian, 1848, *Dibolia cryptocephala* (Koch, 1803), *Euluperus pinicola* (Duftschmid, 1825), *Phyllotreta* sp., *Cassida* sp., *Aphthona* sp., *Luperus* sp.). Consequently, 71 barcode sequences were successfully generated, representing 57 species (Table 1). During generated barcodes validation, morphological and molecular identifications were congruent in all but six cases. Incongruence arose for one of the following two reasons. The first reason was that the best molecular match corresponded to the same species as the morphological identification, but sequence identity or similarity values were below the selected 96% threshold. This included *Cryptocephalus ocellatus* Drapiez, 1819, which matched *C. ocellatus* with 95.01% similarity on BOLD and 94.26% identity on GenBank (E-value < 1 × 10^−20^); *Clytra novempunctata* Olivier, 1808, matching *C. novempunctata* with 93.43% similarity on BOLD; *Clytra atraphaxidis* (Pallas, 1773), matching *C. atraphaxidis* with 94.3% similarity on BOLD and 92.24% identity on GenBank (E-value < 1 × 10^−20^); and *Longitarsus nanus* (Foudras, 1860), matching *L. nanus* with 92.16% similarity on BOLD and 91.93% identity on GenBank (E-value < 1 × 10^−20^). The second reason was that the best molecular match corresponded to a species different from that indicated by morphological identification. For example, sequences of *Altica carduorum* Guérin-Méneville, 1858 matched multiple *Altica* species (including *A. engstromi* (J. Sahlberg, 1893), *A. ampelophaga* Guérin-Méneville, 1858, *A. quercetorum* Foudras, 1860, *A. longicollis* (Allard, 1860), and *A. lythri* (Aubé, 1843) with identity and similarity values ranging from 97.0% to 98.66%. In addition, *Cryptocephalus pygmaeus* Fabricius, 1792 showed its best match with *C. vittatus* Fabricius, 1775 in both BOLD (99.48% similarity) and GenBank (99.36% identity; E-value < 1 × 10^−20^), whereas a match with *C. pygmaeus* itself was retrieved only as a secondary best hit with identity and similarity values ≤ 97% (E-value < 1 × 10^−20^). Several *Longitarsus* sequences, as well as those of *Psylliodes brisouti* Bedel, 1898, matched reference sequences of species other than those recognized by morphological identification under the selected threshold. However, in all cases, at least one reference sequence congruent with the morphological identification was present, showing the same level of identity/similarity as the alternative species. In total, 10 sequences representing eight species had no matches in reference databases above the selected threshold. All of these correspond to species for which no barcode sequences were previously available in BOLD or GenBank, including two species that are likely new to science (Table 1). Among the last two, one *Dibolia* species, matching *Dibolia rugulosa* Redtenbacher, 1849 sequences with identity and similarity of about 91% (GenBank E-value < 1 × 10^−20^) and one *Cassida* species matching *Cassida pannonica* Suffrian, 1844, *Cassida inopinata* (Sassi & Borowiec, 2006), and *Cassida vibex* Linnaeus, 1767, with less than 93.7% sequence similarity and identity (GenBank E-value < 1 × 10^−20^).

### New record for Albanian chrysomelid fauna

This study documents 12 new species records for Albanian fauna (Table 1). Most of these belong to Galerucinae: Alticitae (six species), followed by Cryptocephalinae: Clytrini (three species), while Cryptocephalinae: Cryptocephalini, Galerucinae: Galerucinae, and Eumolpinae: Adoxini are each represented by a single species. Among them, *Bromius obscurus* (Linnaeus, 1758) was collected in eight individuals from *Salix* sp., a plant not previously reported as a host for this species. The specimens were found in a forest clearing with sparse trees and shrubs, surrounded by xeric grassland. In the same clearing, two other species newly recorded for Albania, *Crepidodera lamina* (Bedel, 1901) and *C. nitidula* (Linnaeus, 1758), were collected on *Populus* sp. *Psylliodes brisouti* Bedel, 1898, *Neocrepidodera pereirolii* (Kutschera, 1860), *Cheilotoma musciformis* (Goeze, 1777), and *Calomicrus circumfusus* (Marsham, 1802) were recorded in the xeric grasslands surrounding this clearing. Additional individuals of *C. circumfusus*, along with some of the other newly recorded species, were also found in xeric grasslands with scattered trees and shrubs, including *Rosa* sp., *Rubus* sp., *Crataegus* sp., and *Quercus* spp. The only two species newly recorded for Albania that were found in different habitats were *Cryptocephalus nitidus* (Linnaeus, 1758), collected in a hygrophilous grassland, and *Longitarsus nanus* (Foudras, 1860), collected in a grassland beneath olive trees. In addition to new species records, three further taxa belonging to the genera *Cassida*, *Aphthona*, and *Dibolia* are considered highly likely to represent species new to science. However, all are represented by single specimens. For *Aphthona* sp. and *Dibolia* sp., the limited material collected precludes definitive taxonomic confirmation, as additional specimens are required for thorough evaluation. In contrast, the *Cassida* sp. exhibits morphological characters clearly distinct from those of all described species in the genus and is currently under formal description (Sassi pers. comm.). Moreover, a total of seven *Luperus flaviceps* individuals were collected from localities in Albania different from that of the two specimens reported by Bezděk and Sekerka (2024) (Fig. 1D, Suppl. material 1).

### Redescription

#### Luperus flaviceps

Taxon classificationAnimaliaColeopteraChrysomelidae

Apfelbeck, 1912

B207F220-F1E9-5577-B798-6B2699E7085A

##### Type locality.

Bosnia and Herzegovina, Pale near Sarajevo.

##### Type material examined (via high-quality images of type specimens).

Bosnia and Herzegovina • 1♀, 1 ♂, syntypes; [Sarajevo region], Pale, “bei Saraj.” [near Sarajevo]; V. Apfelbeck leg.; National Museum of Bosnia and Herzegovina, Sarajevo; • 3♀♀, paratypes; [Sarajevo region], Pale, “bei Saraj.” [near Sarajevo]; V. Apfelbeck leg.; National Museum of Bosnia and Herzegovina, Sarajevo.

##### Additional material examined.

Albania • 5♀♀, 1♂; Korçë County, Valamara mountain; alt. ~900 – ~1670 m; 25 Jun. 2025; M. Montagna and G. Magoga leg. • 1♀; Korçë County, Pepellash; alt. 1104 m; 27 Jun. 2025; M. Montagna and G. Magoga leg. • 1♀, 1♂; Korçë County, Radanj env., Lengatices Valley; [40°12'43.4"N, 20°37'08.9"E]; 2 Jun. 2013; P. Vonička leg.; det. J. Bezděk; collection of J. Bezděk, Brno, Czech Republic.

##### Morphology.

Body length. Male 3.9 mm; females 4.5–5.0 mm.

**Figure 3.**
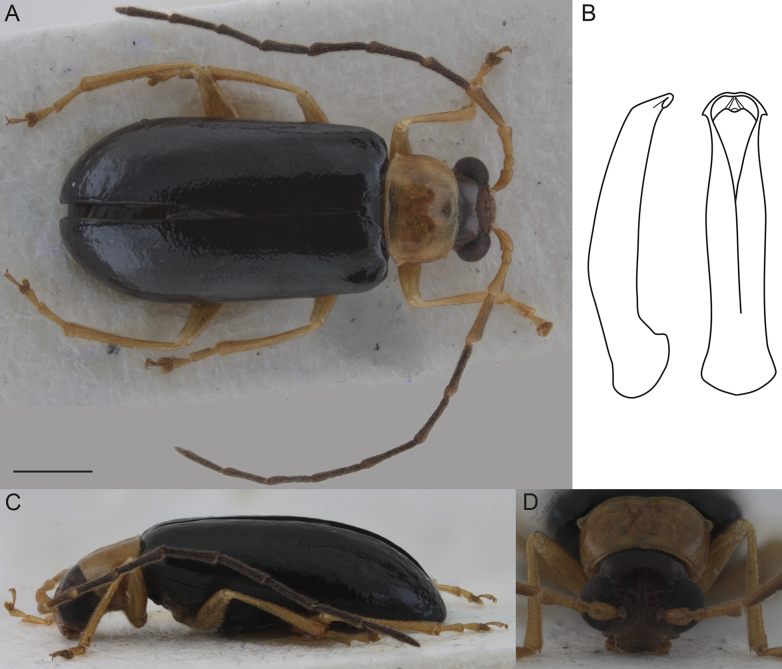
*Luperus flaviceps*, male. **A**. Dorsal view; **B**. Aedeagus drawing; **C**. Lateral view; **D**. Frontal view. Scale bar: 0.7 mm.

**Figure 4.**
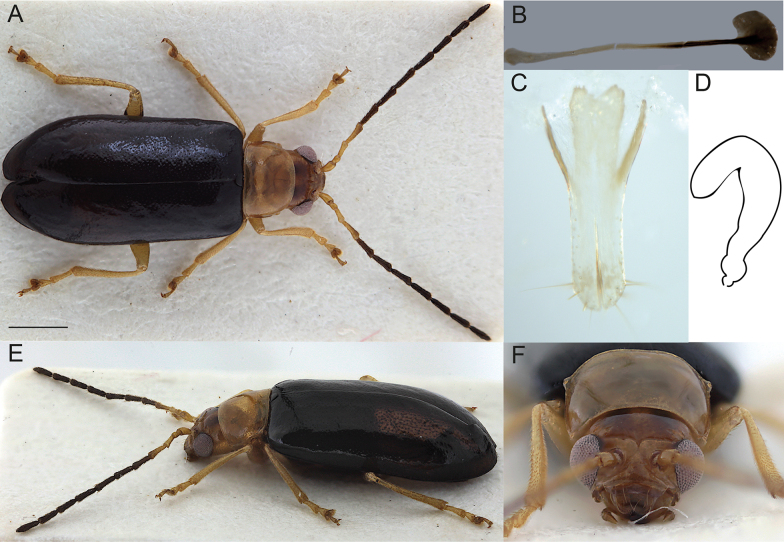
*Luperus flaviceps*, female. **A**. Dorsal view; **B**. Tignum; **C**. Gonocoxite; **D**. Spermatheca; **E**. Lateral view; **F**. Frontal view. Scale bar: 0.85 mm.

***General habitus***. Body elongate, weakly convex, glabrous and shiny. Elytra and ventral surface of body black, contrasting with pale yellow pronotum, head, and legs (Figs 3, 4).

***Head***. Slightly narrower than pronotum; in males, width across eyes exceeds that across pronotum. In males darkened, especially along inner margins of eyes; in females, entire head yellow, although frons may sometimes be slightly darker. Eyes large and prominent. Frontal ridge broad, weakly to moderately convex. Frontal calli large, moderately elevated and wide, smooth and shining, separated from each other and from frons by a deep furrow. Vertex smooth. Antennae filiform: in males slightly longer than body; body length equal to combined length of antennomeres I–X, antennomere XI extending beyond body length; in females distinctly shorter, reaching about ^3^/_4_ of body length. Antennomeres I–III pale, IV slightly darkened ventrally, V–XI darkened.

***Pronotum***. Nearly rectangular, weakly convex, 1.5 times wider than long. Surface smooth and glabrous, with sparse, very fine punctures; lateral margins slightly rounded; all margins bordered. Anterior and posterior angles protruding.

***Scutellum*** subtriangular, glabrous, and shining.

***Elytra***. Elongate, parallel-sided, 0.75 times as long as the body and 1.85 times as long as wide; uniformly black, with smooth lustre; punctuation fine and irregular. Humeral calli distinct; apex rounded. Epipleura rather broad in the basal quarter, rapidly tapering by basal third of elytra, and reduced to a narrow margin in apical half of elytra.

***Legs***. Entirely pale, including femora, tibiae, and tarsi; 3^rd^ tarsomere slightly darker. Legs slender, covered with short, fine pale setae. Each claw with a small basal tooth.

***Ventral side***. Black, finely punctate, sparsely pubescent. Last abdominal ventral sternite of male with deep, drop-shaped impression.

**Sexual dimorphism**. Females larger and more robust than males, with proportionally shorter head and antennae. Head in females typically uniformly yellow, but vertex may be slightly darkened in some individuals; such darkening is weaker and less extensive than in males.

***Aedeagus***. Relatively slender and elongate; median lobe with nearly parallel lateral margins, slightly narrowed basally in lateral view. Apex bifid, with apical margins curved ventrally and laterally, forming a pair of recurved apical teeth. Distinct longitudinal groove present on ventral surface, beginning near apical third and widening uniformly towards apex, merging with contour of apical processes (Fig. 3B).

##### Distribution.

Albania, Bosnia and Herzegovina, Greece (Beenen 2024).

##### Differential diagnosis.

This species is closely related to *Luperus doeberli* Vela & Bastazo, 2017 from south-eastern Turkey, sharing a similar bicoloured pattern. However, *L. flaviceps* can be distinguished by its generally larger body size (3.9–5.0 mm vs 4.3 mm in *L. doeberli*), differences in antennal coloration (antennomeres I–IV pale vs I–VI in *L. doeberli*), more strongly protruding pronotal angles and a distinctly margined anterior side of the pronotum (vs very weakly protruding angles and inconspicuous anterior margin). *Luperus flaviceps* differs from other related European *Luperus* mainly in having the yellow fore part of the head (in males) or entirely yellow head (in females), black elytra without metallic sheen or with only a faint lustre, and legs entirely pale. It is separated from *L. pyrenaeus* Germar, 1824 by the yellow head (vs entirely black) and larger size (3.9–5.0 mm vs. 3.6–4.6 mm), and from *L. caucasicus* Weise, 1879 and *L. margaritae* Lopatin, 1990 by the entirely pale legs (vs at least hind femora basally darkened) and larger body (starting from 3.9 mm vs 3.2–3.7 mm).

##### Molecular identification.

Two DNA barcode sequences (MEDLB1010-26 and MEDLB1011-26) are provided for the molecular identification of this species, one obtained from a male and one from a female specimen; both were collected on Valamara Mountain.

## Discussion

This study contributes to the knowledge of the Albanian chrysomelid fauna by providing geographic records for 81 species in the country (Figs 1, 2, Suppl. material 1), 12 of which have never been recorded in Albania before. In the 14 years between the Palaearctic catalogues of Löbl and Smetana (2010) and Bezděk and Sekerka (2024), collection campaigns and revisions of historical collections of Chrysomelidae have added only nine species records for Albania, indicating that the new data presented here represent a valuable addition to the country’s faunistic knowledge. The new findings largely align with the biogeographical framework proposed by Warchałowski (1976), who described the Western Balkan leaf-beetle fauna as predominantly shaped by Mediterranean influences. The detection of taxa with Balkan, South-Eastern European, or Eastern Mediterranean distributions supports this interpretation. At the same time, many of the newly recorded species are widespread European taxa, suggesting that the updated faunal composition primarily reflects previously overlooked elements rather than the recognition of a distinct biogeographical pattern. Nevertheless, the discovery of 12 new country records together with three potentially undescribed taxa (genus *Cassida*, *Dibolia* and *Aphthona*) demonstrates that the Albanian chrysomelid fauna remains insufficiently explored and that its diversity is still underestimated. For two of these, *Dibolia* sp. and *Aphthona* sp., additional specimens are needed to confirm their status as new species. While no barcode sequence was obtained for *Aphthona* sp., a barcode was successfully generated for *Dibolia* sp. This sequence differs from those of all species expected to occur in Albania according to Bezděk and Sekerka (2024) that are represented in the BOLD system by at least one barcode. The collected individual is a male, and the aedeagus morphology differs from the one of the externally most similar species, *D. depressiuscula* Letzner, 1847 and *D. foersteri* Bach, 1859. Molecularly, the most similar barcode (~91% similarity) corresponds to *D. rugulosa*, which, however, is morphologically very distinct from the Albanian specimen. Regarding *Cassida*, the clear morphological differences observed in both external characters and spermatheca shape in the collected individual, compared to the closest species, those in the *C. vibex* group (Sassi and Borowiec 2006), suggest that it represents a new species for science (currently under description). Molecularly, the closest species with available barcode sequences in BOLD and GenBank are *C. pannonica*, *C. inopinata*, and *C. vibex*, with < 93.7% sequence similarity, further supporting its distinctiveness.

This work also contributes to generating reference barcode sequences for the molecular identification of Albanian chrysomelid species, as well as chrysomelid species occurring both in Albania and other countries. In particular, the 71 barcode sequences generated, representing 57 species, constitute the first DNA barcode dataset for Albanian Chrysomelidae. Among these, sequences of eight species represent new taxon records for public reference databases. Besides the two undescribed taxa, four of these species have distributions largely restricted to the Balkan Peninsula or the eastern part of the Western Palearctic (i.e. *Pachybrachis limbatus* (Menetries, 1832), *Aphthona parnassicola* Heikertinger, 1944, *Luperus flaviceps*, *Phyllobrotica adusta* (Creutzer, 1799)), whereas two other species (*Cassida atrata* Fabricius, 1787 and *Cassida seladonia* Gyllenhal, 1827) show broader distributions across much of Europe (Bezděk and Sekerka 2024). The barcode sequences generated in this study were rigorously validated within an integrative framework, using the BOLDCOI animal database as a reference. The few instances of incongruence between molecular and morphological identification should not be interpreted as errors in morphological species identification, but rather as arising from multiple factors. Some discrepancies result from the use of a fixed nucleotide similarity threshold to differentiate intra- and interspecific variation (even though a permissive threshold was applied in this study), which may not be suitable for all taxa, as its effectiveness depends on their evolutionary histories and the geographic distances between collection localities (Meyer and Paulay 2005; Magoga et al. 2018). Other cases are associated with known errors in chrysomelid barcode reference data or with taxa for which COI-based molecular identification is known to be problematic (e.g. *Longitarsus* and *Altica*; Magoga et al. 2018; Salvi et al. 2020).

Finally, this study provides a detailed redescription of *L. flaviceps*. This rare species was originally described from Bosnia and Herzegovina and, for a long time, was known exclusively from that country (Gruev 2005). More recently, it was also reported from Greece (Beenen 2010) and Albania (Beenen 2024). During the collection campaigns of this study, multiple individuals of *L. flaviceps* were recorded in Albania from two distinct localities, both different from the single locality previously known for the species in the country. Because the original description of this species was undetailed and lacked information on the aedeagus, the availability of multiple specimens enabled a more detailed assessment of the species’ morphology. In the redescription, morphological characters absent from the original description are documented, including sexual dimorphism and aedeagal morphology. In addition, high-quality photographs of male and female specimens and drawings of the aedeagus are provided (Figs 3, 4). These, in combination with the information and images presented by Vela and Bastazo (2017), should provide a clear and comprehensive characterization of this rare species. To further facilitate species identification, DNA barcode sequences have also been generated.

## Conclusions

This study underscores the value of targeted collecting campaigns in underexplored regions, such as Albania, for advancing our knowledge of chrysomelid diversity. Even relatively short-term campaigns, like the one presented here, can produce significant outcomes, including new national records, potentially undescribed species, and important updates to species distributions. Furthermore, the publication of reference DNA barcodes and species occurrence data in global repositories such as BOLD and GBIF greatly enhances the accessibility of chrysomelid biodiversity information and supports a wide range of further research. Finally, revisiting poorly known taxa with modern techniques, as demonstrated here for *L. flaviceps*, helps to fill critical gaps in taxonomic knowledge.

## Supplementary Material

XML Treatment for Luperus flaviceps

## Additional information

### Artificial Intelligence (AI) use

Regarding the use of AI in the preparation of this manuscript, the authors declare the following:

Parts of the text of the manuscript were linguistically refined with the assistance of the artificial intelligence tool ChatGPT; however, all scientific content, including conceptualization, data generation and analysis, interpretation and visualization of results and substantive text writing, derives exclusively from the authors’ work.

### Author ORCIDs

G. Magoga orcidLi https://orcid.org/0000-0002-0662-5840 nk

A. Andreeva https://orcid.org/0009-0006-4000-5609

P. Costagliola https://orcid.org/0009-0009-9486-3862

M. Cardinale https://orcid.org/0009-0004-9438-0338

M. Brunetti https://orcid.org/0000-0003-0817-2942

M. Montagna https://orcid.org/0000-0003-4465-7146

### Data availability

All of the data that support the findings of this study are available in the main text or Supplementary Information.

## Supplementary materials

Supplementary material 1 Occurences Data type xlsx Explanation note **table SS1**. Localities where chrysomelid species were recorded in Albania. For each species, the subfamily is reported, together with the number of individuals per collection locality, geographic coordinates (latitude and longitude), elevation, and collection date. https://binary.pensoft.net/file/1721486This dataset is made available under the Open Database License (http://opendatacommons.org/licenses/odbl/1.0/). The Open Database License (ODbL) is a license agreement intended to allow users to freely share, modify, and use this Dataset while maintaining this same freedom for others, provided that the original source and author(s) are credited. Giulia Magoga, Alevtina Andreeva, Paola Costagliola, Marilù Cardinale, Matteo Brunetti, Matteo Montagna
